# An Efficient and Self-Adapted Approach to the Sharpening of Color Images

**DOI:** 10.1155/2013/105945

**Published:** 2013-11-18

**Authors:** Lih-Jen Kau, Tien-Lin Lee

**Affiliations:** Department of Electronic Engineering & Graduate Institute of Computer and Communication Engineering, National Taipei University of Technology, No. 1 Section 3, Chung-Hsiao E. Road, Taipei 10608, Taiwan

## Abstract

An efficient approach to the sharpening of color images is proposed in this paper. For this, the image to be sharpened is first transformed to the *HSV* color model, and then only the channel of *Value* will be used for the process of sharpening while the other channels are left unchanged. We then apply a proposed edge detector and low-pass filter to the channel of *Value* to pick out pixels around boundaries. After that, those pixels detected as around edges or boundaries are adjusted so that the boundary can be sharpened, and those nonedge pixels are kept unaltered. The increment or decrement magnitude that is to be added to those edge pixels is determined in an adaptive manner based on global statistics of the image and local statistics of the pixel to be sharpened. With the proposed approach, the discontinuities can be highlighted while most of the original information contained in the image can be retained. Finally, the adjusted channel of *Value* and that of *Hue* and *Saturation* will be integrated to get the sharpened color image. Extensive experiments on natural images will be given in this paper to highlight the effectiveness and efficiency of the proposed approach.

## 1. Introduction

The technique of image sharpening is widely applied in a variety of image processing systems, for example, medical image processing, electronic printing, as well as industrial applications such as defect inspections. In these applications, we may want to highlight the transitions or discontinuities in intensity so that the image details of interest can be easily observed. Besides, let us consider the procedures in most of the image processing systems. Usually, the first step is to convolve the sampled image with a low-pass filter so that the noise interference during the sampling process can be removed. Though the problem of noise interference can be solved, however, the quality of the original image can be degraded since image details, for example, edges or boundaries, are blurred by the low-pass filtering process. To conquer this problem, we have to sharpen or emphasize pixels around edges or boundaries but keep the overall intensity unchanged so that the details of the original image can be recovered. To attain the goal of edge sharpening in an image, a so-called panchromatic sharpening (pan-sharpening) technique is widely applied to highlight the details in multiband images such as remote-sensing images captured by satellites [1, 2]. For this, a high-resolution panchromatic image and the lower-resolution multispectral image are merged together to create a single high-resolution or the so-called pan-sharpened color image [1, 2]. On the other hand, the noise suppression can be an annoying problem during the process of edge enhancement. Therefore, a technique combining kernel regression and local homogeneity is proposed in [3]. The image to be sharpened is first filtered with kernel regression, and then the local homogeneity computation is introduced for further smoothing so that the algorithm is effective regarding noise reduction and edge enhancement [3].

In certain cases, the sharpening of an image is accomplished in conjunction with image contrast enhancement [4–12]. In such kind of approaches, the statistics of the image to be sharpened is usually first obtained, and then the equalization of histogram will be performed. Histogram equalization (HE) has been proved to be simple and effective in contrast enhancement. However, it tends to change the mean brightness of the image to the middle level of the permitted range and hence is not very suitable for consumer electronic products, where preserving the original brightness is essential to avoid unnatural look and visual artifacts [4]. To conquer this problem, a brightness preserving histogram equalization with maximum entropy (BPHEME) approach is proposed in [4]. The BPHEME tries to find the target histogram that maximizes the entropy under the constraints that the mean brightness is fixed so that not only the image can be enhanced, but also the original brightness can be preserved as well [4].

The use of an intensity distribution of the whole image is the major cause of visual artifacts in conventional HE. Therefore, some of the researches propose the use of a so-called subregion or subimage HE [6–11]. In [6], the image to be enhanced is first convolved with a Gaussian filter to get smoothed intensity values, and then the transformation function is applied for histogram equalization. With the process of convolving with the Gaussian filter, the transformation function used is not based on the intensity of the pixels only, but the values of the neighboring pixels are also considered [6]. In [8], a recursive subimage HE is developed by iteratively dividing the histogram based on median rather than mean values so that the brightness can be preserved to better extend than previous histogram partitioning methods. A contrast enhancement method using dynamic range separate HE (DRSHE) is proposed in [9]. The DRSHE first separates the dynamic range of histogram into several parts and resizes the gray scale range of each part based on individual area ratio, and then intensities of histogram in each part are uniformly redistributed in resized gray scale range so that unintended changes in brightness can be suppressed [9]. In [10], an edge-preserving contrast enhancement and a multihistogram equalization method are proposed. By utilizing the human visual system, the image to be enhanced is decomposed into segments, resulting in an efficient correction of nonuniform illumination. Additionally, a quantitative measure of image enhancement is also proposed [10]. In [11], an adaptive image equalization algorithm is proposed. The histogram distribution is first synthesized by Gaussian mixture model, and the intersection points of the Gaussian components are used to partition the dynamic range of the image into subintervals. The contrast equalized image is generated by transforming the gray levels in each subinterval according to the dominant Gaussian component and the cumulative distribution function of the subinterval with a weight proportional to the variance of the corresponding Gaussian component. The algorithm is free of parameter setting for a given dynamic range of the enhanced image [11]. A fuzzy logic-based HE (FHE) is proposed in [7]. The fuzzy histogram is first computed based on fuzzy set theory, and then the fuzzy histogram is divided into two subhistograms based on the median value of the original image. Finally, the two subhistograms are equalized independently to get a brightness preserved and contrast enhanced image.

In [12], the Laplace filter is first applied so that the strength of the discontinuity in the image to be processed can be evaluated. After that, a Laplace filter is used again to highlight discontinuity with smaller strength while a Gaussian filter is applied to suppress discontinuity with larger strength. Finally, the contrast will be enhanced by using a proposed adaptive HE approach to get a better visual perceptual quality [12]. In [5], an edge-weighted contrast enhancement algorithm is proposed. The image to be enhanced is first convolved with a median filter to get a low-pass filtered image. Meanwhile, the original image is also processed by a weighted threshold histogram equalization (WTHE) approach to get a rudimentary enhanced image. Finally, the Sobel operator is applied to the original image to be enhanced to get a couple of weights for the low-passed filtered image as well as the rudimentary enhanced image so that the two images can be merged together to get the final enhanced image. In these histogram-equalized approaches, all the pixels are adjusted, in addition to the intensity as well as the characteristics of the original image changes [4–12].

In [13], a content-adaptive algorithm is proposed for the sharpening of images. By extracting the length of lines in the image to be sharpened, the content characteristic as well as the increment or decrement magnitude to be added to the original image can be determined automatically. In [13], regions with artifacts will be sharpened more, while that of with natural objects will be less sharpened [13]. In [14], color image to be sharpened is first converted to *YIQ* or *CIELAB* color space, and then the method of unsharp masking and fuzzy morphological sharpening will be used to adjust the intensity of pixels around boundaries [14]. In [15], a fuzzy logic approach is applied for the sharpening as well as the enhancement of local contrast so that the problem of noise-sensitive in conventional linear unsharp masking technique can be avoided. Aiming to find the additive magnitude automatically, a Grey system-based approach is proposed in [16]. In [16], the maximal additive magnitude is first obtained by using a Grey prediction mechanism with global statistics of the image to be sharpened, and then a portion of the maximal additive magnitude will be used for the sharpening process based on the local statistics of the pixel to be adjusted.

In [17], an edge enhancement approach is proposed by using image fusion technique. The image to be processed is first convolved with low-pass and high-pass filters, where the filter parameters are determined based on the histogram of the image to be processed. After that, the filtered images are adjusted based on the statistics of the filtered images and then fused together to get an edge enhanced image [17]. A Sobel operator-based approach to the sharpening of edges in a grey scale image is proposed in [18]. The image to be sharpened is first processed by a Gaussian low-pass filter to get a blurred image, and then the Sobel operator is applied to find the edges in the image as well as the gradient of the pixel to be processed. In [18], the gradient around a pixel is quantized to one of a set of four predefined angles. After that, a nonmaximum suppression and the so-called *Hysteresis thresholding* approach will be applied for nonedge pixel and salt-and-pepper noises removal, respectively. Finally, the thin and smooth edges in the image can be obtained after these processes.

In this paper, we propose an efficient approach to the sharpening of color images that can adapt itself to the statistics of the image to be sharpened. The image to be sharpened is first transformed to* HSV* color space, and then only the channel of* Value* will be used for the later process of sharpening while that of* Hue* and Saturation are left unchanged. It is noted that the channel of* Value*, a grey-scale image that records the luminance of the image, possesses and exhibits the most important information for human visual perception. Therefore, a high-resolution color image can be obtained by combining low-resolution color bands with the high-resolution luminance band. To perform edge sharpening for the channel of *Value*, we first determine a maximal additive magnitude Δ by using global statistics of the image to be sharpened. After that, we apply a proposed simple edge detector to find discontinuities in the channel of* Value*. It is noted that only those pixels detected as around edges or boundaries will be adjusted for the sharpening purpose, and those nonedge pixels in the channel of *Value* are kept unaltered so that the content information in the original image can be retained. In the proposed approach, a low-pass filtering process will be applied after the edge detection process so that isolated pixels, for example, salt and pepper noise, can be removed and will not be regarded as around an edge. After that, the intensity of the pixels in the channel of *Value* which are detected as around an edge or boundary will be adjusted to highlight the discontinuity. The increment or decrement magnitude *δ*_*x*_, that is to be added to the edge pixel *x* to be adjusted, is a portion of the maximal additive magnitude Δ and is determined by the local characteristics of the pixel to be adjusted. That is, the proposed algorithm can adapt itself first to the global statistics of the image to be sharpened and then the local statistics of the pixel to be adjusted. Finally, the sharpened *Value* channel will be combined with the channel of *Hue* and *Saturation* to create the sharpened color image. As we will see in the experiments, the proposed approach can have a very distinct intensity transition for pixels around edges or boundaries in the sharpened images, which demonstrates the usefulness of the proposed approach.

The rest of the paper is organized as follows.  Section 2 gives a quick review on the commonly used Red, Green, and Blue (*RGB*) color model as well as the *Hue*, *Saturation*, and *Value* (*HSV*) color model.  Section 2 also gives an introduction on the color format transformation between *RGB* and *HSV* model. The detailed descriptions on the proposed color image sharpening algorithm can be addressed in Section 3, where the proposed approach will be given in a step-by-step manner. Extensive experimental results by using subjective as well as objective evaluation on the proposed approach will be given in Section 4. Finally, a concluding remark is given in Section 5.

## 2. The *RGB* and* HSV* Color Space

Among all the color image models in image processing systems, the *RGB* color space is one of the most widely used format for image representation. However, human visual perceptual system is most sensitive to the changes of intensity value. That is, the Luminance component brings the most information for human visual perception [19]. Therefore, the* RGB* color model that assumed equal importance on the three components of* Red*,* Green*, and *Blue* does not meet the sensitivity of human visual perception and is not very suitable to be used for the sharpening purpose.

**Figure 1:**
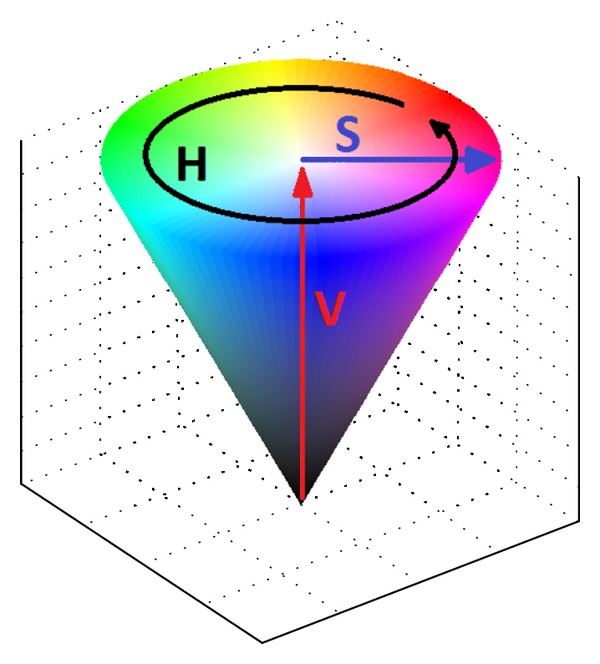
The commonly used *HSV* color model.

In the proposed algorithm, we use another widely applied color space, the so-called* HSV* color model instead of the *RGB* color model, for the sharpening of color images [19]. The* HSV* color model, which rearranges the geometry of *RGB* model in a cylindrical coordinate, is shown in Figure 1. As can be seen in Figure 1, the* HSV* color model which takes the shape of a cone is usually referred to as “hexcone model". In the* HSV* color model, the component “*Hue*" is what we normally think of as color. It is usually represented by an angle between 0° and 360°, which indicates the attribute of a visual sensation according to which an area appears to be similar to one of the perceived colors, for example, red, yellow, green, and blue, or to a combination of them. On the other hand, the component “*Saturation*” is a measure of how different a color appears from a grey of the same lightness. The value of *Saturation* is usually represented with a value from 0 to 1. When the value is 0, the color is grey, and when the value is 1, the color is a primary color. A faded color is due to a lower saturation level, which means that the color contains more grey. The component “*Value*” describes the brightness of the color and varies with color saturation. It is usually represented with a value from 0 to 1. When the value is 0, the color will be totally black. With the use of *Hue*, *Saturation*, and *Value* as components, the characteristic of *HSV* color model is more intuitive and perceptually relevant to human visual system than that of the Cartesian representation of *RGB* model [19].

In this paper, a color image that is originally represented by* RGB* color format should be first transformed to *HSV* color space and then sent for the process of image sharpening with the proposed approach. After being transformed to the* HSV* color space, only the channel of* Value* will be used for the processing of image sharpening. Finally, the adjusted* Value* channel will be combined with that of *Hue* and* Saturation* to get the sharpened color image.

### 2.1. *RGB* to *HSV* Color Transformation

In this subsection, we introduce how a color image that is originally represented by *RGB* format can be transformed to the *HSV* color format. Before the color space conversion, the three components in *RGB* format, that is, *r*, *g*, and *b*, should be normalized to a value between 0 and 1, and then the three components in *HSV* color space, that is, *h*, *s*, and *v*, are calculated according to the following equations:

(1) $$\begin{array}{c} h=\{\begin{array}{cc} \text{undefined}, & \text{if}\;\max=\min;\\ 60^{\circ }\times \frac{g-b}{\max-\min}+0^{\circ }, & \text{if}\;\max=r,g\geq b;\\ 60^{\circ }\times \frac{b-r}{\max-\min}+120^{\circ }, & \text{if}\;\max=g;\\ 60^{\circ }\times \frac{r-g}{\max-\min}+240^{\circ }, & \text{if}\;\max=b;\\ 60^{\circ }\times \frac{g-b}{\max-\min}+360^{\circ }, & \text{if}\;\max=r,\;g<b. \end{array} \end{array}$$

(2) $$\begin{array}{c} s=\{\begin{array}{cc} 0, & \text{if}\;\max=0;\\ \frac{\max-\min}{\max}=1-\frac{\min}{\max}, & \text{otherwise}. \end{array} \end{array}$$

(3) $$\begin{array}{c} v=\max. \end{array}$$

As can be seen in (1)–(3), the range of *h* is a value between 0° and 360°, while that of *s* and *v* both is between 0 and 1. Besides, we can find in the first condition of (1) and the second condition of (2) that the* Hue* is not defined and the *Saturation* would be 0 if the value of the three components in *RGB* color format is identical. The pixel under conversion turns to be a grey pixel and only the component of *Value* is meaningful when this happens.

### 2.2. *HSV* to *RGB* Color Transformation

In this paper, the adjusted channel of *Value* will be combined with that of *Hue* and *Saturation* to get the final sharpened color image. In this subsection, we introduce how a color image that is represented by *HSV* format can be transformed back to the *RGB* color format. The three components, (*r*, *g*, *b*), in *RGB* color space are determined according to equations (4)–(7). During the conversion, we first determine two indexes called *h*_*i*_ and *f* according to (4) and (5), respectively:

(4) $$\begin{array}{c} h_{i}=\left\lfloor \frac{h}{60}\right\rfloor \text{ mod }6, \end{array}$$

(5) $$\begin{array}{c} f=\frac{h}{60}-h_{i}. \end{array}$$

After the indexes *h*_*i*_ and *f* are determined, a set of parameters called *p*, *q*, and *t* are then calculated according to the following equations:

(6) $$\begin{array}{c} p=v\times \left(1-s\right),\\ q=v\times \left(1-f\times s\right),\\ t=v\times \left(1-\left(1-f\right)\times s\right). \end{array}$$

Finally, the color vector (*r*, *g*, *b*) is given by

(7) $$\begin{array}{c} \;\left(r,g,b\right)=\{\begin{array}{cc} \left(v,t,p\right), & \;\text{if}\;h_{i}=0;\;\\ \left(q,v,p\right), & \;\text{if}\;h_{i}=1;\;\\ \left(p,v,t\right), & \;\text{if}\;h_{i}=2;\;\\ \left(p,q,v\right), & \;\text{if}\;h_{i}=3;\;\\ \left(t,p,v\right), & \;\text{if}\;h_{i}=4;\;\\ \left(v,p,q\right), & \;\text{if}\;h_{i}=5.\; \end{array} \end{array}$$

## 3. Proposed Color Image Sharpening Algorithm

In this section, the proposed color image sharpening algorithm will be introduced in detail with a step-by-step manner.

### 3.1. Color Space Transformation

 In the proposed approach, the first step is to convert the image that is originally represented by *RGB* color format to *HSV* color space by using the formulas from (1) to (3).

### 3.2. Determine the Maximal Additive Magnitude Δ

 Since the human visual perception system is most sensitive to the changes of intensity values [19], only the channel of *Value* will be used for the process of image sharpening after the color space conversion from *RGB* to *HSV*. That is, what we have to do is to get a sharpened *Value* channel so that a sharpened color image can be obtained by combining the adjusted *Value* channel with the original *Hue* and *Saturation* channels.

During the process of the sharpening of* Value* channel, we just treat the* Value* channel as if it is a grey-scale image. To highlight the discontinuity, an additive magnitude should be imposed on those edge pixels to be adjusted. We know that a larger additive magnitude can have a better sharpening result; however, it can also lead to the saturation of intensity around edge pixels. Aiming to find the maximal additive magnitude Δ automatically, we determine in this paper the value of Δ with the global statistics of the channel *V*, that is, the* Value* channel, to be sharpened so that the condition of oversharpening can be avoided.

To do this, we first find out the *Min*⁡, *Max*⁡, Mid, and Avg of the channel *V* by using the following equations:

(8) $$\begin{array}{c} \mathrm{Max}=\text{maximum}\left(V\right),\\ \mathrm{Min}=\text{minimum}\left(V\right),\\ \text{Mid}=\frac{\mathrm{Max}+\mathrm{Min}}{2},\\ \text{Avg}=\frac{\sum_{i=1}^{M}\sum_{j=1}^{N}V_{i,j}}{M\times N}, \end{array}$$

where *V*_*i*,*j*_ is the intensity of the *Value* channel at position (*i*, *j*) and *M* and *N* are the height and width of the image to be processed, respectively.

To find a suitable additive magnitude Δ that can be widely applied to images to be sharpened so that the discontinuity of an edge or boundary can be highlighted, we find in our extensive experiments that a magnitude of Max*⁄*8 would be a good choice. That is, when an increment or decrement of Max*⁄*8 is imposed on those edge pixels, a noticeable difference before and after the sharpening process can be commonly perceived by human visual system. Moreover, an additive magnitude slightly larger than Max*⁄*8 is required for an image with higher brightness, while an additive magnitude slightly smaller than Max*⁄*8 is enough for an image with lower brightness. We therefore multiply Max*⁄*8 with a correction term Avg/Mid during the selection of the maximal additive magnitude Δ, and the maximal additive magnitude Δ in this paper is determined by

(9) $$\begin{array}{c} \Delta =\frac{\mathrm{Max}}{8}\times \frac{\text{Avg}}{\text{Mid}}, \end{array}$$

where *Max*⁡, Avg, and Mid are global statistics of the image to be sharpened defined in (8).

### 3.3. The Edge Detection Mechanism

**Figure 2:**
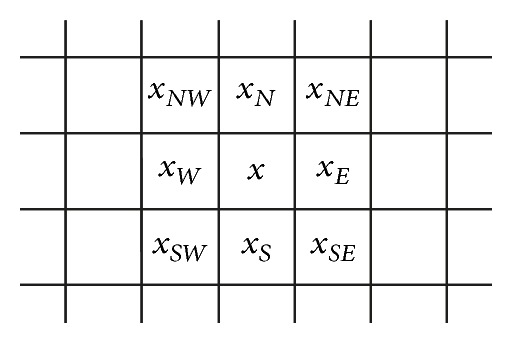
Texture context of the pixel *x* to be sharpened.

 Considering the run-time performance, we propose in this paper a simple yet effective approach to the detection of an edge in the channel of *Value*. We call the proposed edge detection approach the horizontal and vertical differentiator (*HVD* for short). The discontinuity around a pixel *x* can be easily detected by examining the intensity difference between (*x*, *x*_*W*_) and (*x*, *x*_*N*_) (in Figure 2). We then determine if the pixel *x* is around an edge by checking if the first condition of the following equation holds:

(10) $$\begin{array}{c} g\left(x\right)=\{\begin{array}{cc} 1, & \text{if}\;\left|x-x_{W}\right|\geq \theta_{\text{eth}}\;\text{or}\left|x-x_{N}\right|\geq \theta_{\text{eth}}\\ 0, & \text{otherwise}, \end{array} \end{array}$$

where *g* is a bi-level output image with 1*s* represented for edge pixels, *x* is the pixel under gradient evaluation, *x*_*W*_ and *x*_*N*_ are defined in Figure 2, and *θ*_eth_ is a predefined threshold which controls the degree of discontinuity that a pixel may be regarded as around an edge. Empirically, a value between 8 to 18 would be a suitable choice for *θ*_eth_.

### 3.4. Low-Pass Filtering for Isolated Pixel Removal

 During the edge detection process, some of the isolated pixels can also be detected as around an edge with the proposed first derivative *HVD* edge detector. Therefore, not only the edge information, but also the salt-and-pepper noise can be incurred in the bi-level image *g* (in (11)) as well. To avoid this problem, a low-pass filtering process is applied so that those isolated pixels can be excluded from being regarded as around an edge. To determine if a pixel *x* is really an edge pixel or not, we just check if the following inequality holds:

(11) $$\begin{array}{c} \sum_{\forall p\in N_{8}\left(x\right)}g\left(p\right)\geq \theta_{\text{Lpf}}, \end{array}$$

where *N*_8_(*x*) means the eight-connected neighbors of pixel *x*, and *θ*_Lpf_ is a predefined threshold between 0 and 8. That is, we check the number of 1*s* of the eight neighbors of *x* in the bi-level image *g* (Figure 2). If it is smaller than a predefined threshold *θ*_Lpf_, *x* is regarded as an isolated point and will be discarded from the list of edge pixel. In this paper, *θ*_Lpf_ is set to be 2. This is due to the fact that the detected number of edge pixel would be at least two with the proposed *HVD* approach if an edge segment is passing through the pixel *x*. Thus, a value of 2 is suitable for *θ*_Lpf_.

### 3.5. Edge Sharpening for *V*-Channel

 In this step, the intensity of those pixels detected as around an edge are adjusted with an increment or decrement to highlight the discontinuity, and that of those nonedge pixels are kept unaltered. To highlight smaller discontinuity but keep the image not to be oversharpened, the additive magnitude has to be adapted to the local statistics of the image. Thus, we first compute the average intensity LocalMean of a small local area including the edge pixel *x* to be adjusted and the eight-connected neighbors of *x* (the nine pixels in Figure 2). We then compare if the intensity of *x* is greater or smaller than the value of LocalMean. If the intensity value of *x* is greater than LocalMean, an increment *δ*_*x*_ will be added to *x*; otherwise, the *δ*_*x*_ will be subtracted from *x*. The additive value *δ*_*x*_ is determined adaptively by

(12) $$\begin{array}{c} \delta_{x}=\{\begin{array}{cc} s\times \Delta \times \left(\frac{x}{\text{LocalMean}}\right), & \text{if}\;\;x<\text{LocalMean};\\ s\times \Delta \times \left(\frac{\text{LocalMean}}{x}\right), & \text{otherwise}, \end{array} \end{array}$$

where *s* is a scaling factor between 0 and 1 that controls the degree of sharpness, and Δ is obtained in (9). Actually, the term inside the bracket of (12) is for local adaptation. Obviously, a larger value of *δ*_*x*_ which is close to Δ will be obtained if the intensity of *x* is also close to the value of LocalMean, meaning that a larger additive magnitude will be used for an edge with smaller discontinuity and vice versa. The sharpened intensity value $\hat{x}$ of *x* is then given by

(13) $$\begin{array}{c} \hat{x}\{\begin{array}{cc} x-\delta_{x}, & \text{if}\;x<\text{LocalMean};\\ x+\delta_{x}, & \text{otherwise}. \end{array} \end{array}$$

### 3.6. *HSV* to* RGB* Transformation

 During the final step, the adjusted *Value* channel, a high-resolution channel now is combined with the low-resolution channels of *Hue* and *Saturation* to get the sharpened or high-resolution color image. The sharpened color image is now in the format of *HSV*, and can be transformed to the *RGB* color space by using (4) to (7) if needed.

**Figure 3:**
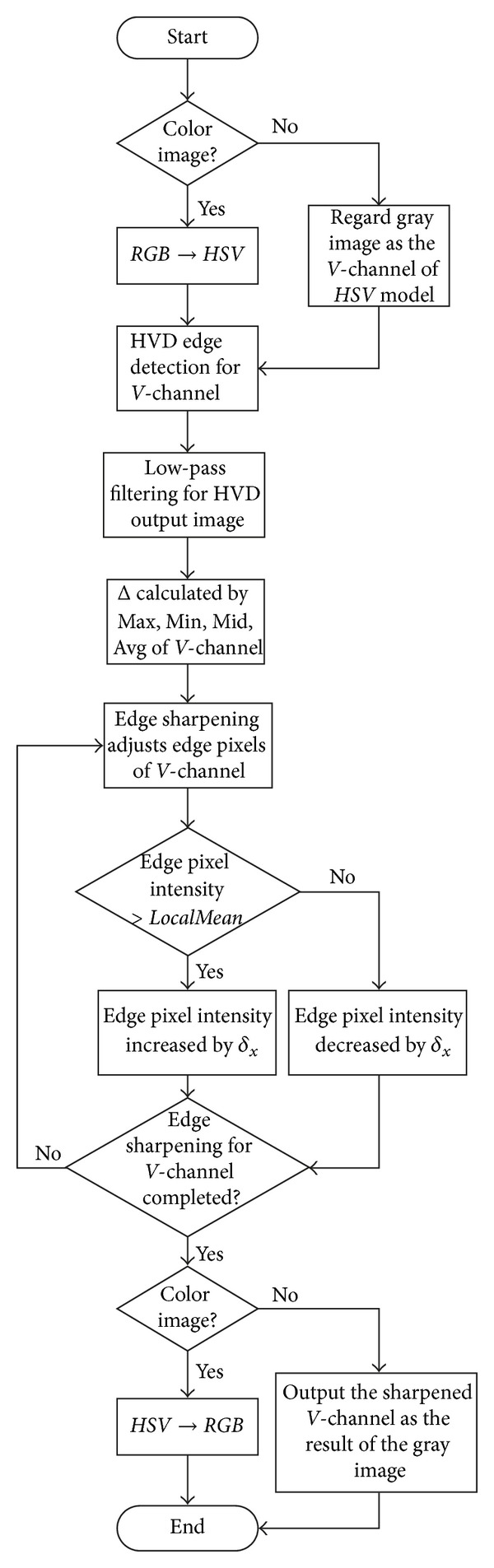
Procedure for the proposed color image sharpening algorithm.

To summarize, we show in Figure 3 the detailed procedure of the proposed color image sharpening algorithm. As can be seen in Figure 3, the proposed algorithm can also be applied to grey-scale image directly by regarding the grey-scale image as the* Value* channel of a color image.

## 4. Experiments

In this section, the effectiveness of the proposed approach is to be evaluated through twelve test images. Among which three of them, that is, the image “Neck”, “Moon surface”, and “Goldhill”, are 8 bit grey-scale image rather than in color format. That is, we also evaluate the effectiveness of the proposed sharpening algorithm on grey-scale images. Besides, all the nine color images are originally stored in* RGB* format.

First of all, the proposed algorithm will be applied to the test image “House” in a step-by-step manner so that the function of each block in Figure 3 can be presented. After that, a subjective evaluation on the sharpening result will be performed, and then an objective evaluation on the quality will be given. During the objective evaluation process, we compare the sharpened result with the original image and check to see if most of the image content can be preserved after the edge sharpening process. Finally, a complexity analysis on the proposed approach will be given by using the operation count to highlight the efficiency of the proposed algorithm.

### 4.1. Step-by-Step Functional Block Evaluation

**Figure 4:**
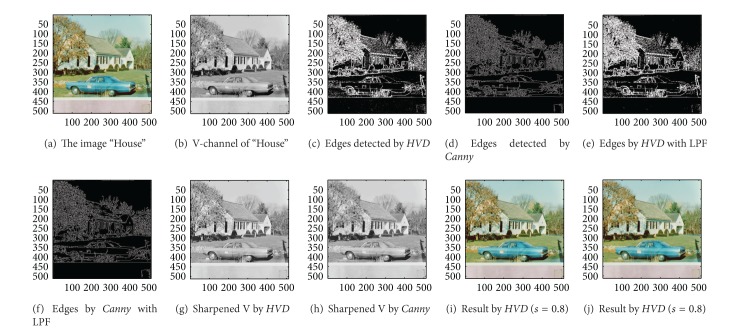
*θ*_eth_ = 13 for *HVD*. (a) The image “House” (512 × 512 color image). (b) *V*-channel of “House". (c) Edges detected by *HVD*. (d) Edges detected by *Canny*. (e) Edges detected by *HVD* with LPF. (f) Edges detected by *Canny* with LPF. (g) Sharpened *Value* channel by *HVD* with LPF. (h) Sharpened *Value* channel by *Canny* with LPF. (i) Sharpened result by *HVD* with scaling factor 0.8. (j) Sharpened result by *Canny* with scaling factor 0.8.

We first show in Figure 4 the proposed algorithm in a step-by-step manner with the test image “House” to demonstrate the superiority as well as the functionality of each block of the proposed approach. The original image “House” is shown in Figure 4(a).  Figure 4(b) shows the *Value* channel of the image “House”, and Figure 4(c) shows the edges of Figure 4(b) which is picked out by using the proposed *HVD* edge detector. For comparison purpose, we also show in Figure 4(d) the edges of Figure 4(b) detected by using the well-known *Canny* operator [20]. As can be seen in Figure 4(d), the edge detected by using the *Canny* operator is fine and subtle when compared with that of obtained by using the proposed *HVD* edge detector in Figure 4(c).

As we are using the first derivative for edge detection of an image, some of the isolated pixels can be regarded as around an edge which results in salt- and pepper noise in the bi-level image (10), for example, in the bottom of Figure 4(c). However, after applying the low-pass filtering process (in (11)), the salt-and-pepper noise can be removed successfully (as in Figure 4(e)). In this step, we also apply the same low-pass filtering process to the edges detected by *Canny* operator (Figure 4(d)), and the filtered result is shown in Figure 4(f). As can be seen in Figures 4(d) and 4(f), it does not make much difference before and after the low-pass filtering process due to a series of complex procedures in *Canny* edge detection process, for example, the Gaussian filtering process for noise reduction, the nonmaximum suppression as well as the hysteresis thresholding, and so forth.

After the low-pass filtering process, those edge pixels in the *Value* channel will be sharpened to highlight the discontinuities. The sharpened *Value* channel by using the proposed approach with *HVD* as well as the *Canny* operator is shown in Figures 4(g) and 4(h), respectively. Finally, the sharpened *Value* channel in conjunction with the unaltered channel of *Hue* and *Saturation* will be combined and transformed back to the *RGB* color format. The sharpened color images obtained by using the proposed *HVD* edge detector and that of by using the *Canny* operator are shown in Figures 4(i) and 4(j), respectively. As can be seen in Figure 4(i), the contour of the house as well as that of the car has become quite conspicuous when compared with that of in the original image (Figure 4(a)). Moreover, the result obtained by using the proposed *HVD* edge detector also exhibits a better visual quality than that of by using the *Canny* operator (Figures 4(i) and 4(j)). For this, recall that the purpose of image sharpening is to highlight the discontinuities for pixels around edges or boundaries, and a better visual perception on the texture or contour can be obtained if pixels on both sides of an edge can be adjusted simultaneously, that is, to increase the sharpness on both sides of an edge simultaneously. However, when we look into the edges detected by using the proposed *HVD* edge detector and that of by *Canny* operator in Figures 4(e) and 4(f), we find that the width of an edge detected by using the *Canny* operator is subtle and would be only one-pixel in most of the cases, meaning that only pixels in one side of an edge will be adjusted. Therefore, the discontinuity can not be as distinct as that of by using the* HVD* edge detector under the same scaling factor *s*.

### 4.2. Subjective Performance Evaluation

 In this subsection, the sharpened result by using the proposed approach to a set of twelve test images in Table 1, including the first nine color images and last three grey images, will be given and evaluated in a subjective manner.

**Figure 5:**
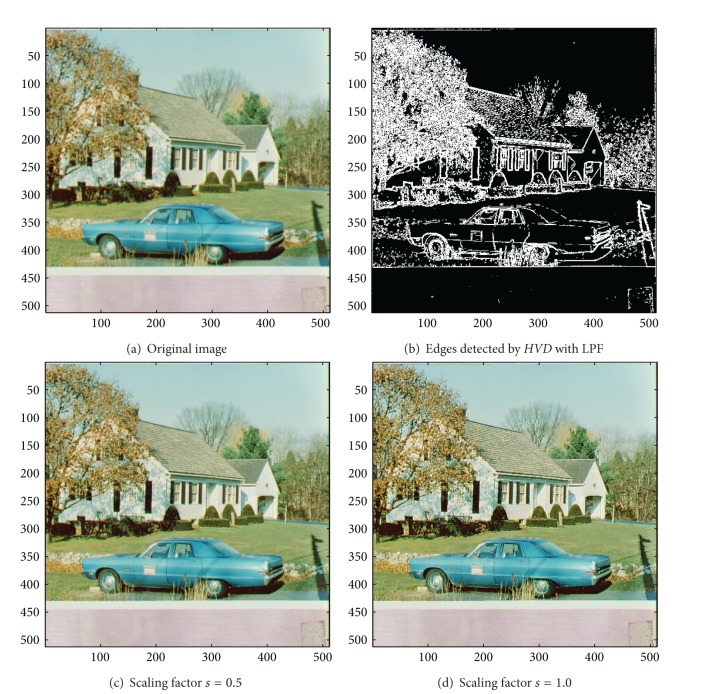
*θ*_eth_ = 13 for *HVD*. (a) The image “House” (512 × 512 color image). (b) Edges detected by *HVD* with LPF. (c and d) Sharpened results by *HVD* with scaling factors 0.5 and 1.0, respectively.

For the test image “House”, we also show in Figures 5(c) and 5(d) the results obtained by using different scaling factors (*s* = 0.5 in Figure 5(c) and *s* = 1.0 in Figure 5(d)). As can be seen in Figure 5(d), a larger scaling factor *s* usually can have a better sharpening result for human visual perception, and the oversharpening phenomenon does not take place when *s* = 1.

**Figure 6:**
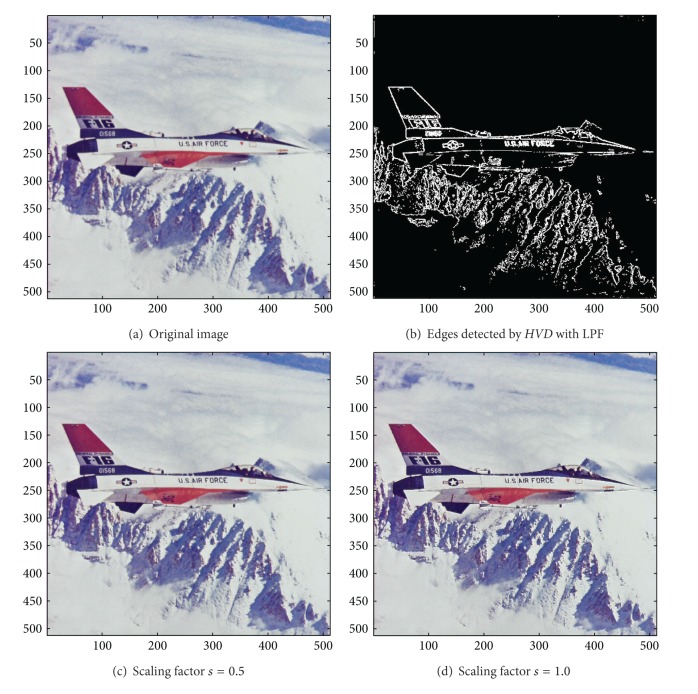
*θ*_eth_ = 15 for *HVD*. (a) The image “F-16” (512 × 512 color image). (b) Edges detected by *HVD* with LPF. (c and d) Sharpened results by *HVD* with scaling factors 0.5 and 1.0, respectively.

**Figure 7:**
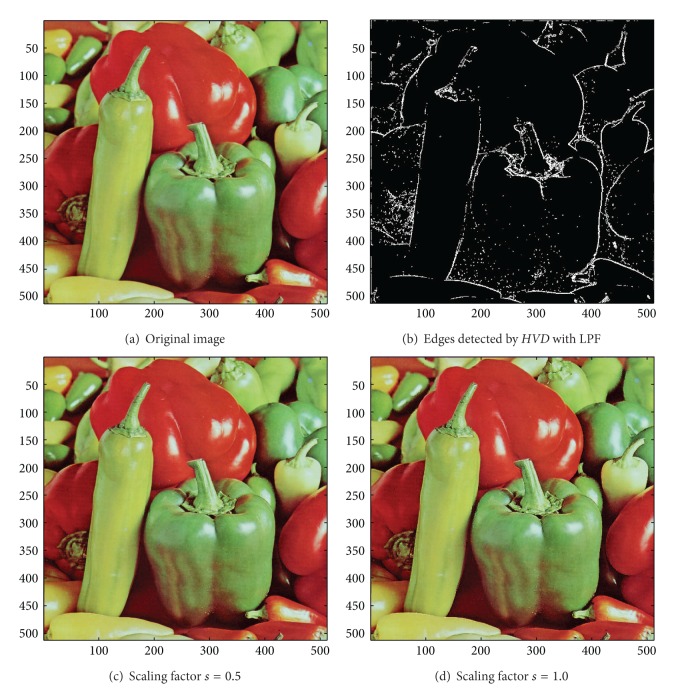
*θ*_eth_ = 22 for *HVD*. (a) The image “Peppers” (512 × 512 color image). (b) Edges detected by *HVD* with LPF. (c and d) Sharpened results by *HVD* with scaling factors 0.5 and 1.0, respectively.

**Figure 8:**
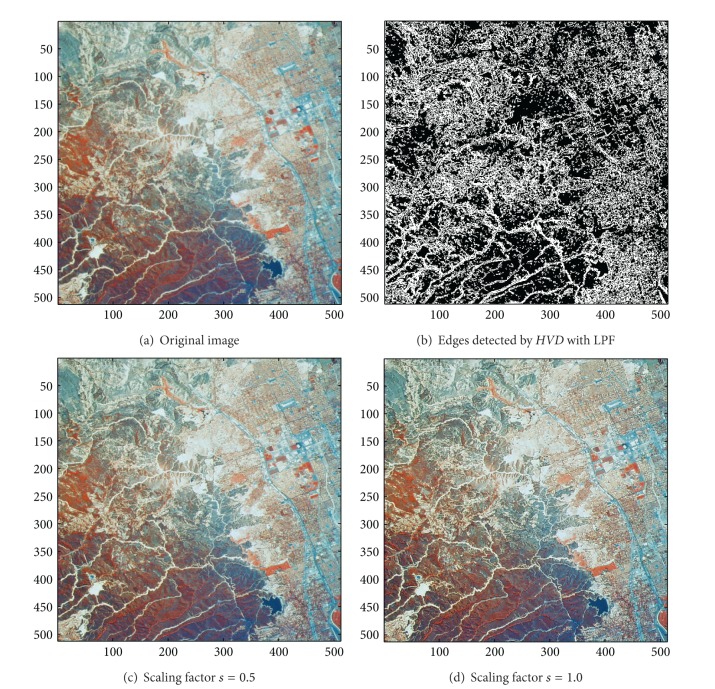
*θ*_eth_ = 15 for *HVD*. (a) The image “Woodland Hills, CA” (512 × 512 color image). (b) Edges detected by *HVD* with LPF. (c and d) Sharpened results by *HVD* with scaling factors 0.5 and 1.0, respectively.

**Figure 9:**
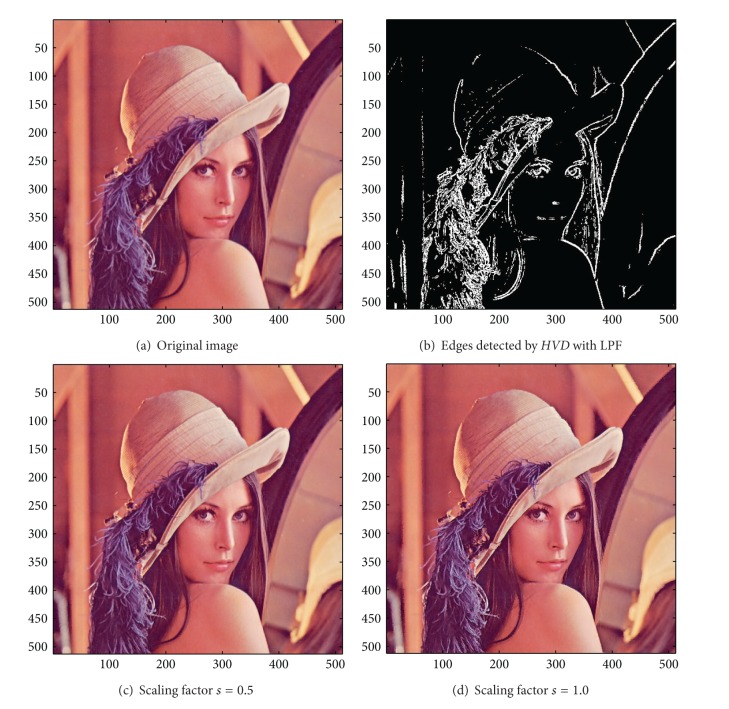
*θ*_eth_ = 16 for *HVD*. (a) The image “Lena” (512 × 512 color image). (b) Edges detected by* HVD* with LPF. (c and d) Sharpened results by *HVD* with scaling factors 0.5 and 1.0, respectively.

**Figure 10:**
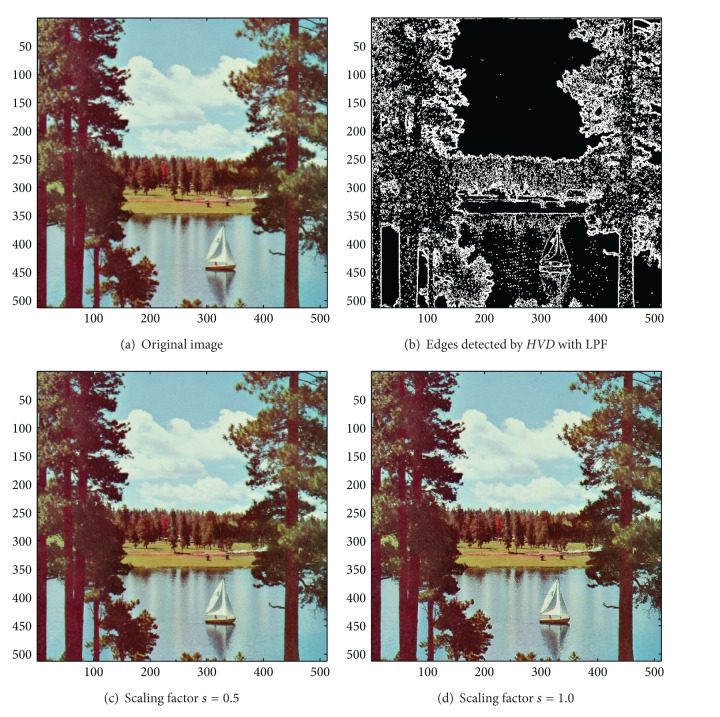
*θ*_eth_ = 16 for *HVD*. (a) The image “Sailboat on lake” (512 × 512 color image). (b) Edges detected by *HVD* with LPF. (c and d) Sharpened results by *HVD* with scaling factors 0.5 and 1.0, respectively.

**Figure 11:**
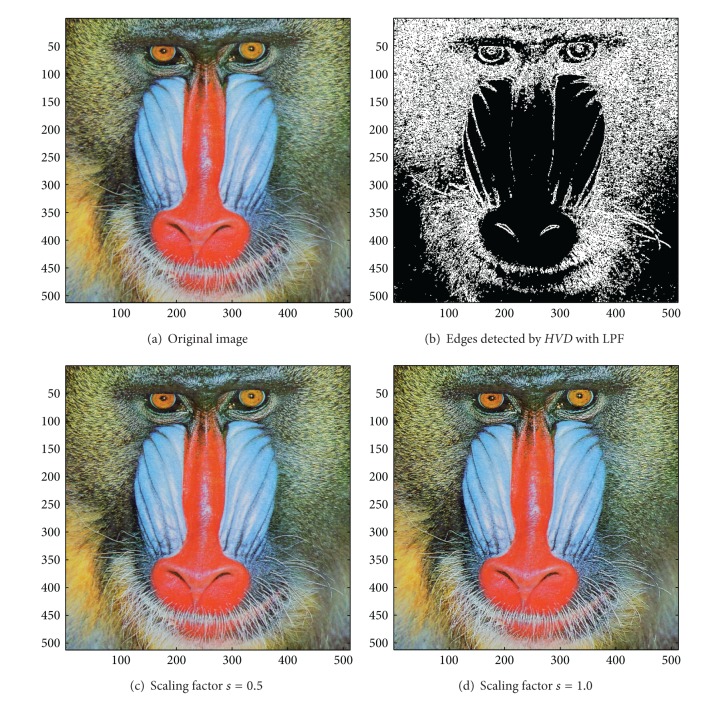
*θ*_eth_ = 16 for *HVD*. (a) The image “Baboon” (512 × 512 color image). (b) Edges detected by *HVD* with LPF. (c and d) Sharpened results by *HVD* with scaling factors 0.5 and 1.0, respectively.

**Figure 12:**
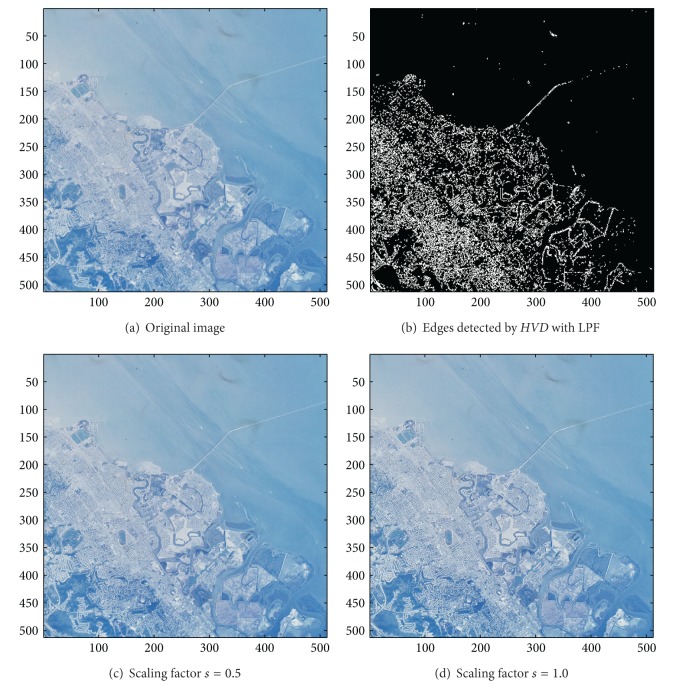
*θ*_eth_ = 9 for *HVD*. (a) The image “Foster City, CA” (512 × 512 color image). (b) Edges detected by *HVD* with LPF. (c and d) Sharpened results by *HVD* with scaling factors 0.5 and 1.0, respectively.

**Figure 13:**
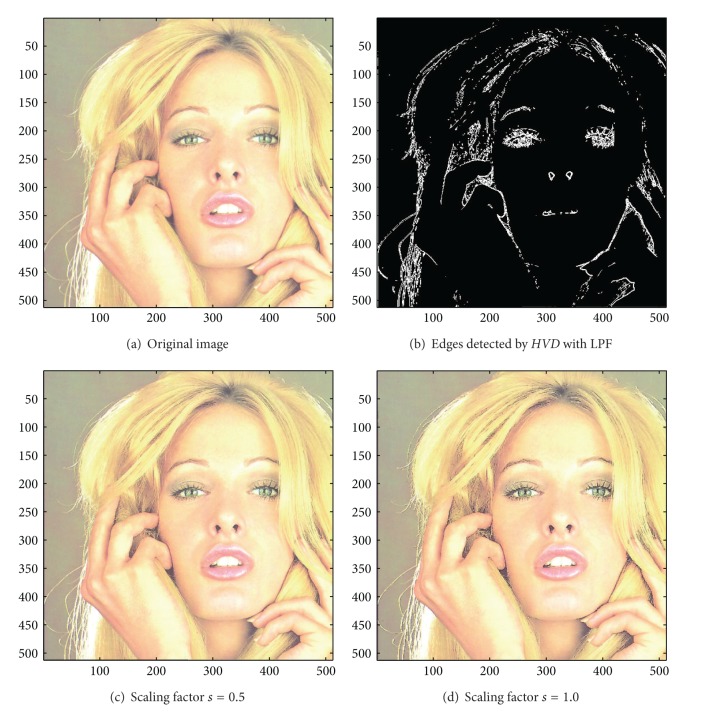
*θ*_eth_ = 18 for *HVD*. (a) The image “Tiffany” (512 × 512 color image). (b) Edges detected by *HVD* with LPF. (c and d) Sharpened results by *HVD* with scaling factors 0.5 and 1.0, respectively.

In addition, we also show from Figures 6 to 13 the sharpened results obtained by using the proposed approach to eight other color test images. As can be seen in Figures 6(c) and 6(d), the contour of the plane, the text on the plane, the pilot, and the mountain all have a very good visual quality when compared with the original image in Figure 6(a). For the test image “Peppers” in Figure 7, we can see in Figures 7(c) and 7(d) a very distinct contour around peppers and around the stalk of these peppers after the sharpening process when compared with the original image in Figure 7(a). For the test image “Woodland Hills, CA”, an aerial image, in Figure 8, we can see that the contour of the mountain, lake, roads and buildings are quite distinct in Figures 8(c) and 8(d) after the proposed sharpening process.  Figure 9 shows the results of the well-known test image “Lena”. As can be seen in Figures 9(c) and 9(d), the contour around her eyes and the contour of the hair have become very conspicuous after the sharpening process.  Figure 10 shows the results of the test image “Sailboat on lake”. When compared with the original image in Figure 10(a), a more conspicuous contour can be obtained for the sailboat, the waves of the lake, and the forest after the sharpening process (Figures 10(c) and 10(d)). The results of the test image “Baboon” are shown in Figure 11. As can be seen in Figures 11(c) and 11(d), the contour around the eyes, and the beard or moustache of the baboon have become quite obvious when compared with the original image in Figure 11(a).  Figure 12 shows the results of the test image “Foster City, CA”, an aerial image. A remarkable contour around the buildings, the bridges, and the roads can be obtained after the proposed sharpening process (Figures 12(c) and 12(d)) when compared with that of in the original image (Figure 12(a)). For the test image “Tiffany”, we show in Figures 13(c) and 13(d) the sharpened results by using the proposed approach. As can be seen in Figures 13(c) and 13(d), a very distinct contour around her eyes and around her fingers can be obtained after the sharpening process when compared with that of the original image in Figure 13(a).

**Figure 14:**
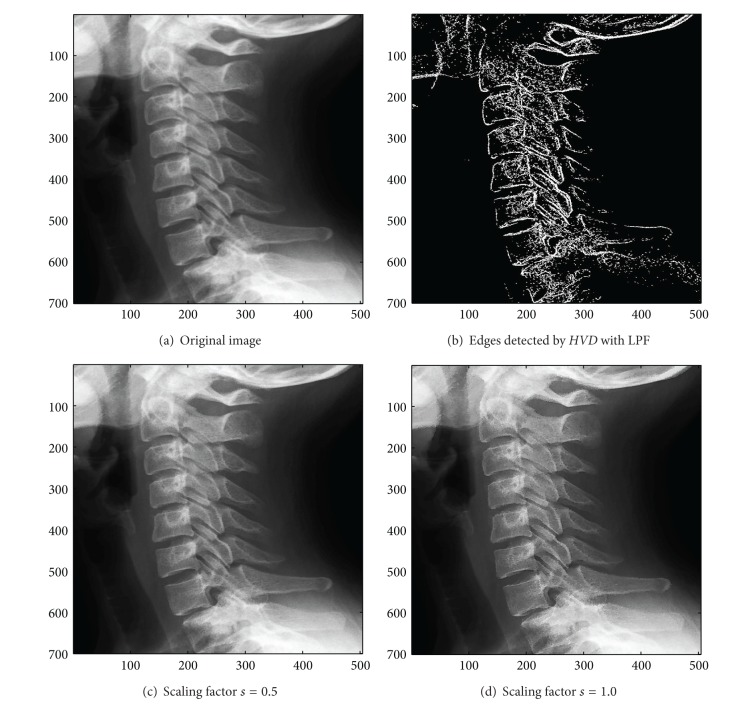
*θ*_eth_ = 7 for *HVD*. (a) The image “Neck” (504 × 700 grey image). (b) Edges detected by *HVD* with LPF. (c and d) Sharpened results by *HVD* with scaling factors 0.5 and 1.0, respectively.

**Figure 15:**
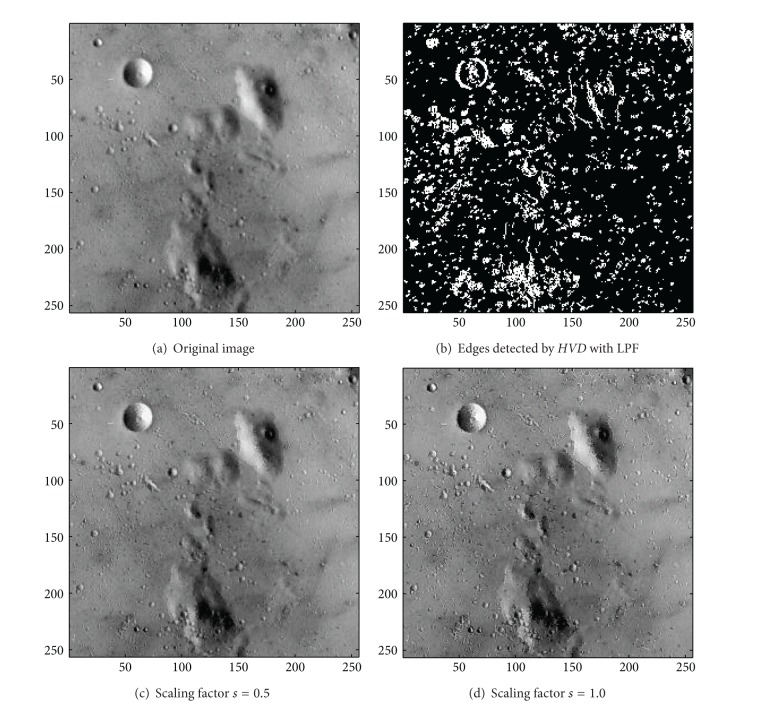
*θ*_eth_ = 16 for *HVD*. (a) The image “Moon surface” (256 × 256 grey image). (b) Edges detected by *HVD* with LPF. (c and d) Sharpened results by *HVD* with scaling factors 0.5 and 1.0, respectively.

**Figure 16:**
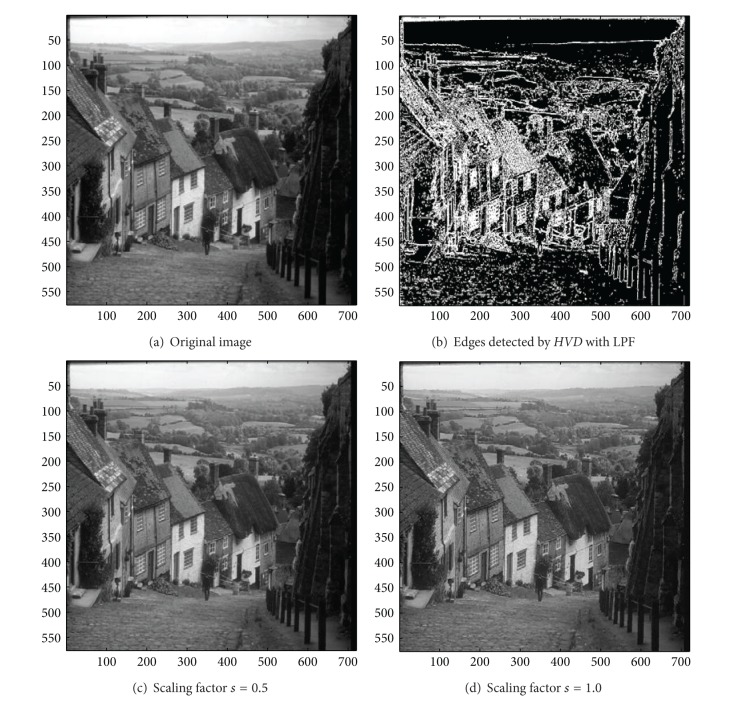
*θ*_eth_ = 10 for *HVD*. (a) The image “Goldhill” (720 × 576 grey image). (b) Edges detected by *HVD* with LPF. (c and d) Sharpened results by *HVD* with scaling factors 0.5 and 1.0, respectively.

In addition to the first nine color test images in Table 1, we also investigate the usefulness of the proposed approach on the three grey-scale test image, that is, the image “Neck”, the image “Moon surface”, and the image “Goldhill”. We first look at the test image “Neck”, a medical image, in Figure 14. As can be seen in Figures 14(c) and 14(d), the contour of the cervical vertebra has become quite obvious after the sharpening process when compared with that of the original image in Figure 14(a). The sharpened results for the test image “Moon surface” are shown in Figures 15(c) and 15(d), respectively. As can be seen in Figures 15(c) and 15(d), those cavities and mounds have become more conspicuous when compared with the original image in Figure 15(a). Finally, for the test image “Goldhill” in Figure 16, the contour of the roof tiles, and the outline of the windows have become more distinct (Figures 16(c) and 16(d)) than that of in the original image (Figure 16(a)).

In this subsection, subjective performance evaluations on several kinds of test images, for example, natural images, aerial images, and medical images have been carried out and verified. In addition, not only color images, but also grey-scale images are evaluated in this part. As can be seen in Figures 5 to 16, the usefulness of the proposed sharpening algorithm can be demonstrated. Moreover, a conspicuous contour can be visually observed without the phenomenon of oversharpening when the scaling factor *s* is selected to be 1, which justifies the selection of the maximal additive magnitude Δ.

### 4.3. Objective Performance Evaluation with PSNR

 In addition to the subjective evaluation on the test images, most of the research applies the *peak signal to noise ratio* (PSNR) as well to evaluate the objective quality of the proposed image sharpening algorithm. That is, we want to make the contour or outline of an image visually conspicuous, but with most of the content information preserved. Therefore, we use the PSNR as a metric to check the difference of an image before and after the sharpening process. The results of the objective performance evaluation obtained by using the proposed approach are listed in Table 1. The maximal additive value Δ for individual test image is also listed in the last column of Table 1. As can be seen in Table 1, the sharpened images can still have a very good PSNR, which indicates that most of the information in the original image can be retained after the sharpening process.

### 4.4. Complexity of the Proposed Algorithm

 In this subsection, a computational complexity analysis is given in the form of a table showing the operation counts in each step during the sharpening process of the proposed approach. The operation counts of the proposed approach are listed in Table 2. The second row of Table 2 indicates the operation counts required to perform the horizontal and vertical difference edge detection. It is noted that the operation counts of low-pass filtering and sharpening are required only for those edge pixels.

Though the edges picked out by using the proposed *HVD* edge detector is not as subtle as that of by using the *Canny* operator, the sharpened image results are satisfactory since pixels on both side of an edge are adjusted simultaneously to make the discontinuities visually more conspicuous. Considering the sharpened image results and the computational complexity, a very good trade-off has been obtained which justifies the superiority of the proposed approach.

## 5. Conclusion

An efficient approach to the sharpening of color images is proposed in this paper. The image to be sharpened is first transformed to the *HSV* color format, and then only the channel of *Value* will be used for the process of sharpening while the other two channels are left unchanged. After that, pixels detected as around edges or boundaries are adjusted to highlight the discontinuity, and those nonedge pixels are kept unaltered. It is noted that the increment or decrement magnitude that is to be added to those edge pixels is determined in an adaptive manner first based on global statistics of the image and then the local statistics of the pixel to be sharpened. With the proposed adaptive approach, the discontinuities can be highlighted while most of the original information contained in the image can be retained. Finally, the adjusted channel of *Value* and that of *Hue* and *Saturation* will be integrated to get the sharpened color image. Extensive experiments on natural images, aerial images, medical image, and grey-scale images with subjective and objective performance evaluations have demonstrated the effectiveness and efficiency of the proposed approach.

## Figures and Tables

**Table 1:** Comparisons of the sharpened images with respect to original images in terms of PSNR.

Image	Dimension	Edge sharpening images						
		(a) *HVD* without LPF			(a) *HVD* with *θ*_LPF_ ≥ 2			Δ
		*s* = 0.5	*s* = 0.8	*s* = 1.0	*s* = 0.5	*s* = 0.8	*s* = 1.0	
House	512 × 512	68.63	59.06	54.59	69.12	59.55	55.08	39
F-16	512 × 512	79.55	69.90	65.42	80.43	70.78	66.30	38
Peppers	512 × 512	86.41	77.02	72.58	88.84	79.49	75.06	41
Woodland Hills, CA	512 × 512	62.27	52.75	48.32	62.90	53.38	48.95	41
Lena	512 × 512	85.90	76.41	72.00	86.73	77.26	72.84	36
Sailboat on lake	512 × 512	73.09	63.48	59.00	73.91	64.30	59.82	36
Baboon	512 × 512	66.30	56.72	52.21	66.66	57.07	52.56	39
Foster City, CA	512 × 512	79.15	69.46	64.96	80.74	71.08	66.59	32
Tiffany	512 × 512	77.31	68.98	65.16	78.32	70.01	66.19	58
Neck	504 × 700	88.76	78.60	73.93	91.03	80.88	76.21	20
Moon surface	256 × 256	76.04	66.40	61.87	78.46	68.83	64.30	31
Goldhill	720 × 576	76.39	66.67	62.14	77.37	67.65	63.12	23
Average		76.65	67.12	62.68	77.87	68.36	63.92	36

“*s*” is scaling factor between 0 and 1.

Low-pass filtering (LPF) and edge sharpening is for edge pixel only.

**Table 2:** Operation counts of the proposed approach.

Operations	APU	MPU/DIV	ABS	COMP
*HVD *	≤2	0	≤2	≤2
Low-pass filtering	7	0	0	1
Edge sharpening	9	4	0	1

*HVD* is required for each pixel.

LPF and edge sharpening is for edge pixel only.
